# The Role of Emotional Intelligence, the Teacher-Student Relationship, and Flourishing on Academic Performance in Adolescents: A Moderated Mediation Study

**DOI:** 10.3389/fpsyg.2021.695067

**Published:** 2021-07-14

**Authors:** María Teresa Chamizo-Nieto, Christiane Arrivillaga, Lourdes Rey, Natalio Extremera

**Affiliations:** ^1^Department of Personality, Assessment and Psychological Treatment, Faculty of Psychology, University of Malaga, Málaga, Spain; ^2^Department of Social Psychology, Social Work, Social Anthropology and East Asian Studies, Faculty of Psychology, University of Malaga, Málaga, Spain

**Keywords:** emotional intelligence, flourishing, academic performance, teacher-student relationship, adolescence

## Abstract

Educational context has an important influence on adolescents’ development and well-being, which also affects their academic performance. Previous empirical studies highlight the importance of levels of emotional intelligence for students’ academic performance. Despite several studies having analyzed the association and underlying mechanisms linking emotional intelligence and academic performance, further research, including both personal and contextual dimensions, is necessary to better understand this relation. Therefore, the purpose of this study was to deepen the understanding of the effect of emotional intelligence has on academic performance, examining the possible mediating role of flourishing and the moderating role of the teacher-student relationship. A convenience sample of 283 adolescents (49.8% female), aged 12–18 years (*M* = 14.42, SD = 1.12), participated in a cross-sectional study by completing self-report questionnaires measuring emotional intelligence (Wong and Law Emotional Intelligence Scale), flourishing (Flourishing Scale), and teacher-student relationship (Inventory of Teacher-Student Relationships) and reported their grades of the previous term on four mandatory subjects in the Spanish education curriculum. Results indicated that flourishing completely mediated the path from emotional intelligence to academic performance and that teacher-student relationship was a significant moderator in this model. Thus, in adolescents with worse teacher-student relationship, the association of emotional intelligence and flourishing was stronger than in adolescents with better teacher-student relationship. In turn, flourishing was positively associated with academic performance. These results suggest that it is crucial to foster better teacher-student relationship, especially in adolescents with low emotional intelligence, and to positively impact their well-being and their academic performance.

## Introduction

During adolescence, development and well-being are influenced by changes in the social, biological, and personal spheres (e.g., familial, educational, or cultural). The educational context is one of the most influential, in which adolescents spend a great amount of time learning new skills and establishing social relationships (Alford, 2017). In this context, the academic performance of students is one of the most essential criteria in evaluating them. Prior research has analyzed how academic performance is predicted by a number of personal and other environmental dimensions (Deighton et al., 2018; Carmona-Halty et al., 2019; Hayat et al., 2020; Zhou et al., 2020). Research shows that emotional intelligence, flourishing, and teacher-student relationships are among the positive resources that promote well-being, psychological adjustment, and academic performance (e.g., Datu, 2018; Lan and Moscardino, 2019; Rey et al., 2019; MacCann et al., 2020).

### Emotional Intelligence and Academic Performance

In the current study, emotional intelligence is conceptualized from the ability model developed by Mayer et al. (2016) and is defined as a mental ability for perceiving, understanding, using, and regulating one’s own and other people’s emotions. Research literature suggests that emotionally intelligent people report better psychological adjustment (e.g., self-esteem, happiness, optimism, social support, and less depression; Lopez-Zafra et al., 2019; Tejada-Gallardo et al., 2020) as well as higher levels of life satisfaction, well-being, and flourishing (Sánchez-Álvarez et al., 2016; Callea et al., 2019; Lopez-Zafra et al., 2019; Salavera et al., 2020). In educational context, previous findings suggest that developing emotional competences may be a useful resource to increase the levels of flourishing and improve psychological adjustment and interpersonal relationships in adolescent population (Rey et al., 2019; Trigueros et al., 2019; Martínez-Martínez et al., 2020). Furthermore, evidence shows that emotional intelligence is moderately associated with students’ academic performance (MacCann et al., 2020; Sánchez-Álvarez et al., 2020). A plausible explanation for this significant link is that emotional intelligent people are better able to manage emotions associated with educational settings (e.g., stress, frustration, or exam anxiety), and this set of abilities also helps by improving the relationships with peers and teachers (MacCann et al., 2020). Thus, recent studies have explored various underlying mechanisms, such as positive emotions, emotional management, or self-directed learning (e.g., Zhoc et al., 2018; Trigueros et al., 2019; MacCann et al., 2020), that might also explain the link between emotional intelligence and academic performance. Moreover, MacCann et al. (2020) have suggested that some key noncognitive qualities, such as emotional intelligence, might impact on academic performance due to the current changes in education (e.g., an increased in group activities or teamwork), which require learning to manage possible peer conflicts, making decisions, or solving problems in a group. Nonetheless, further studies are necessary to deepen the understanding of emotional intelligence-academic performance linkage. In this study, we propose analyzing the possible mediating role of flourishing and the moderating role of teacher-student relationship in the relation between emotional intelligence and academic performance.

### Flourishing as Mediator

Flourishing can be defined as a way “to live within an optimal range of human functioning, one that connotes goodness, generativity, growth, and resilience” (Fredrickson and Losada, 2005, p. 678). Moreover, it has been proposed as an indicator of well-being encompassing the experience of feeling joy, contentment, or happiness in life (i.e., hedonic well-being) as well as having an effective psychological functioning (i.e., eudaemonic well-being; Huppert and So, 2013). In fact, flourishing is related to less burnout and higher levels of health, life satisfaction, and work engagement (Garzón-Umerenkova et al., 2018; Younes and Alzahrani, 2018; Freire et al., 2020; Imran et al., 2020). Although this variable has not been extensively studied in adolescents (Witten et al., 2019), and even less in relation to academic performance, some studies suggest that higher levels of flourishing may contribute to a better academic performance and a greater likelihood of prioritizing academic chores (Datu, 2018; Datu et al., 2020). Moreover, this variable has been proposed as a significant mediator in the relation between several personal resources such as emotional intelligence and psychological adjustment indicators (e.g., suicide risk; Rey et al., 2019). In line with the aforementioned empirical studies and prior research linking emotional intelligence and academic performance (e.g., Datu, 2018; Callea et al., 2019; Rey et al., 2019; Datu et al., 2020), in the present study, we aimed to examine the potential role of flourishing as mediator in the emotional intelligence-academic performance link.

### Teacher-Student Relationship as Moderator

The quality of the teacher-student relationship constitutes an important aspect in adolescents’ development and mental health (Lippard et al., 2018; Wang et al., 2018, 2020). Previous studies have found that a positive and close teacher-student relationship may increase enjoyment in learning and social adjustment, leading to higher satisfaction of psychological needs and increased peer relationships at school, as well perhaps decreasing academic stress and school burnout in students (Bakadorova and Raufelder, 2018; Lan and Moscardino, 2019; Clem et al., 2020; Luo et al., 2020; Romano et al., 2020; Dong et al., 2021). Furthermore, some studies have shown the benefits of positive teacher-student relationship in promoting the development of adolescents’ emotional intelligence (Wang et al., 2020) and buffering negative consequences of stressful situation (e.g., victimization) on psychological security (Jia et al., 2018). Hence, one might tentatively assume that teacher-student relationship might have an interaction effect with emotional intelligence on subjective and psychological well-being (i.e., flourishing).

### The Current Study

Based on these findings and some gaps in the literature about the relation among emotional intelligence, academic performance, flourishing, and teacher-student relationship, the main objective of this study was to examine the underlying mechanisms in the linkage between emotional intelligence and academic performance, analyzing the roles of flourishing and teacher-student relationship by a moderated mediation model. For this, the following hypotheses were proposed: (1) flourishing will mediate the positive effect of emotional intelligence on academic performance and (2) teacher-student relationship will moderate the relation between emotional intelligence and flourishing.

## Materials and Methods

### Participants

A non-random convenience sample was composed of 283 adolescents (50.2% males and 49.8% females), aged 12–18 years (*M* = 14.42, SD = 1.12), from two public secondary schools in the Andalusia region (Spain). The majority of the sample (93.2%) was Spanish. With regard to grade level: 31.1% were in the 2nd year, 37.5% in the 3rd year, and 31.4% in the 4th year of compulsory secondary education.

### Measures

Emotional intelligence was measured using the Wong and Law Emotional Intelligence Scale (WLEIS: Wong and Law, 2002). The WLEIS is a self-report questionnaire containing 16 items that measure four dimensions of emotional intelligence: self-emotion appraisal (e.g., “I have a good sense of why I feel certain feelings most of the time”), other-emotion appraisal (e.g., “I always know my friends’ emotions from their behavior”), use of emotions (e.g., “I always set goals for myself and then try my best to achieve them”), and regulation of emotions (e.g., “I am able to control my temper and handle difficulties rationally”). A global score can be calculated based on these dimensions. Items are answered on a scale from 1 (“totally disagree”) to 7 (“totally agree”) and higher scores indicate higher levels of emotional intelligence. In this study, we used the Spanish version, which has shown adequate validity and reliability (Extremera et al., 2019). As shown in Table 1, our sample’s reliability indexes were excellent (*α* = 0.91; *ω* = 0.92).

**Table 1:** Descriptive statistics, reliability indexes, and correlations for the study variables.

		*M*	SD	*S*	*K*	*α*	*ω*	1	2	3	4
1.	Emotional intelligence	4.899	1.144	−0.463	−0.068	0.91	0.92	-			
2.	Flourishing	43.561	7.492	−0.877	0.560	0.81	0.81	0.640^**^	-		
3.	Teacher-student relationship	2.648	0.567	0.106	−0.549	0.86	0.87	0.409^**^	0.453^**^	-	
4.	Academic performance	5.640	1.742	0.278	−0.479	0.86	0.87	0.155^*^	0.224^**^	0.177^**^	-

*N* = 282; M, mean; *SD*, standard deviation; S, skewness; K, kurtosis; *α*, Cronbach’s alpha; *ω*, McDonald’s omega.

* *p* < 0.05;

** *p* < 0.01.

Flourishing was assessed using the Flourishing Scale (FS: Diener et al., 2010). The FS is a one-dimension self-report questionnaire, which measures several aspects of positive human functioning such as personal competence, positive relationships, and purpose in life. The scale comprised eight items (e.g., “People respect me”) that are answered on a 7-point scale ranging from 1 (“strongly disagree”) to 7 (“strongly agree”), so higher scores indicate higher levels of well-being. We used the Spanish validated version, which shows good validity and reliability (Checa et al., 2018). The internal consistency in this study was good (*α* = 0.81; *ω* = 0.81).

Teacher-student relationship quality was measured using the Inventory of Teacher-Student Relationships (ITSR: Murray and Zvoch, 2011). The ITSR is a student-report measure of three dimensions of teacher-student relationships: trust (e.g., “I trust my teacher”), communication (e.g., “My teacher understands me”), and alienation (e.g., “I get upset easily at school,” reverse scored). The inventory has 17 items measured on a 4-point scale ranging from 1 (“never”) to 4 (“always”). A mean score of all the items was calculated, so higher scores suggest a better teacher-student relationship. The questionnaire was adapted into Spanish following international guidelines for adapting tests (International Test Commission, 2017). First, two researchers independently translated the original English version into Spanish. Second, a third bilingual translator performed the back-translation. In this process, great care was taken to preserve the content expression of the items. Discrepancies were discussed until agreement on the final version was reached. Following data collection, the reliability of the complete scale was analyzed. Results showed good internal consistency (*α* = 0.86; *ω* = 0.87).

Finally, academic performance was assessed using the grades of the previous term (September to December 2020) reported by the students. An average score was calculated based on four mandatory subjects in the Spanish education curriculum: mathematics, geography and history, Spanish language and literature, and foreign language. Global grades were ranged from 1 (“poor”) to 10 (“excellent”) so higher scores indicate better academic performance. The internal consistency of this measure was good (*α* = 0.86; *ω* = 0.87).

### Procedure

The University of Malaga’s Ethical Committee assessed and approved the research protocol of this study (reference number: 62-2016-H). First, two public schools’ administrations were contacted by phone, they were informed of the study’s objectives and procedure, and they were invited to participate in the cross-sectional study. Upon agreement, they signed an informed consent and notified the students’ parents or legal guardians. Following each school’s policy, parents and legal guardians gave their consent on behalf of the students, either in written form or by not expressing dissent. Data were collected at the schools during a routine class session in the presence of a teacher and a research assistant. During this session, students were informed of the objectives of the study and were assured of the anonymity and confidentiality of their responses. Following, instructions to complete the questionnaires were given and all questions were answered. Students voluntarily completed the paper-based questionnaires for approximately 25 min. Data collection was in accordance with current ethical standards (World Medical Association, 2013).

### Data Analysis

Analyses were carried out using JASP 0.13.0.0 and SPSS 23. Cronbach’s alpha and McDonald’s omega indexes were calculated to assess the reliability of the questionnaires. Descriptive statistics and Pearson correlations were estimated. As self-report questionnaires were used to measure all variables, common-method bias was assessed using Harman’s single-factor test (Podsakoff and Organ, 1986). The PROCESS macro for SPSS (Hayes, 2018) was used to estimate the mediating effect of flourishing on the emotional intelligence-academic performance association (model 4). Moreover, model 7 of the same macro was used to test the moderating effect of the quality of teacher-student relationship in the tested mediation model. The assumptions of independence, normality, multicollinearity, and homoscedasticity were tested prior to conducting the analyses (Field, 2013). For the mediation and moderated mediation analyses a bootstrapping method was used to obtain bias-corrected 95% confidence intervals (95% CI) with 5,000 re-samples. An effect was considered as significant if the 95% CI did not contain zero.

## Results

### Preliminary Analyses

Table 1 shows the descriptive statistics, reliability indexes (coefficients Alpha and Omega), and Pearson correlation analyses for the study variables. As shown, the internal consistency of all the questionnaires was satisfactory and all the variables were significantly and positively correlated. Moreover, Harman’s test indicated that there were nine factors with eigenvalues higher than 1 and the first factor accounted for 24.75% of the variance, so common-method bias was not an issue in this study. Lastly, statistical indexes (i.e., Durbin-Watson = 1.506; VIF values < 10) and plot analyses indicated that all regression assumptions were met.

### Mediating Effect of Flourishing

Table 2 presents the results of the mediation analysis. As shown, emotional intelligence was positively associated with flourishing (*p* < 0.001), which was positively related to academic performance (*p* < 0.05). The total effect of emotional intelligence on academic performance (*b* = 0.184, SE = 0.090, *p* = 0.041) was statistically significant. Moreover, the 95% bootstrap CI for the indirect effect (*b* = 0.178, SE = 0.080, 95% CI = 0.035–0.349) did not contain zero, indicating a statistically significant effect. As the direct effect of emotional intelligence on academic performance (*b* = 0.006, SE = 0.116, *p* = 0.958) was not statistically significant, the results suggest that flourishing completely mediated the positive association between emotional intelligence and academic performance. The model accounted for 11.4% of the variance in academic performance.

**Table 2:** Mediating effect of flourishing on the association of emotional intelligence and academic performance.

Predictors	On flourishing				On academic performance			
	*B*	SE	*t*	*p*	*B*	SE	*t*	*p*
Constant	27.214	4.966	5.479	<0.001	10.024	1.508	6.647	<0.001
Gender (cov)	1.085	0.703	1.543	0.123	0.193	0.203	0.952	0.341
Age (cov)	−0.407	0.313	−1.302	0.193	−0.453	0.090	−5.024	<0.001
Emotional intelligence	4.225	0.310	13.603	<0.001	0.006	0.116	0.052	0.958
Flourishing					0.042	0.017	2.385	0.017
*R*^2^	0.423		<0.001		0.133			<0.001
*F*	64.315				10.066			

*N* = 267; *B*, unstandardized coefficient; SE, standard error; cov, covariate; EI, emotional intelligence. Gender was dummy coded (1 = male, 2 = female).

### Moderating Effect of Teacher-Student Relationship

**Figure 1:**
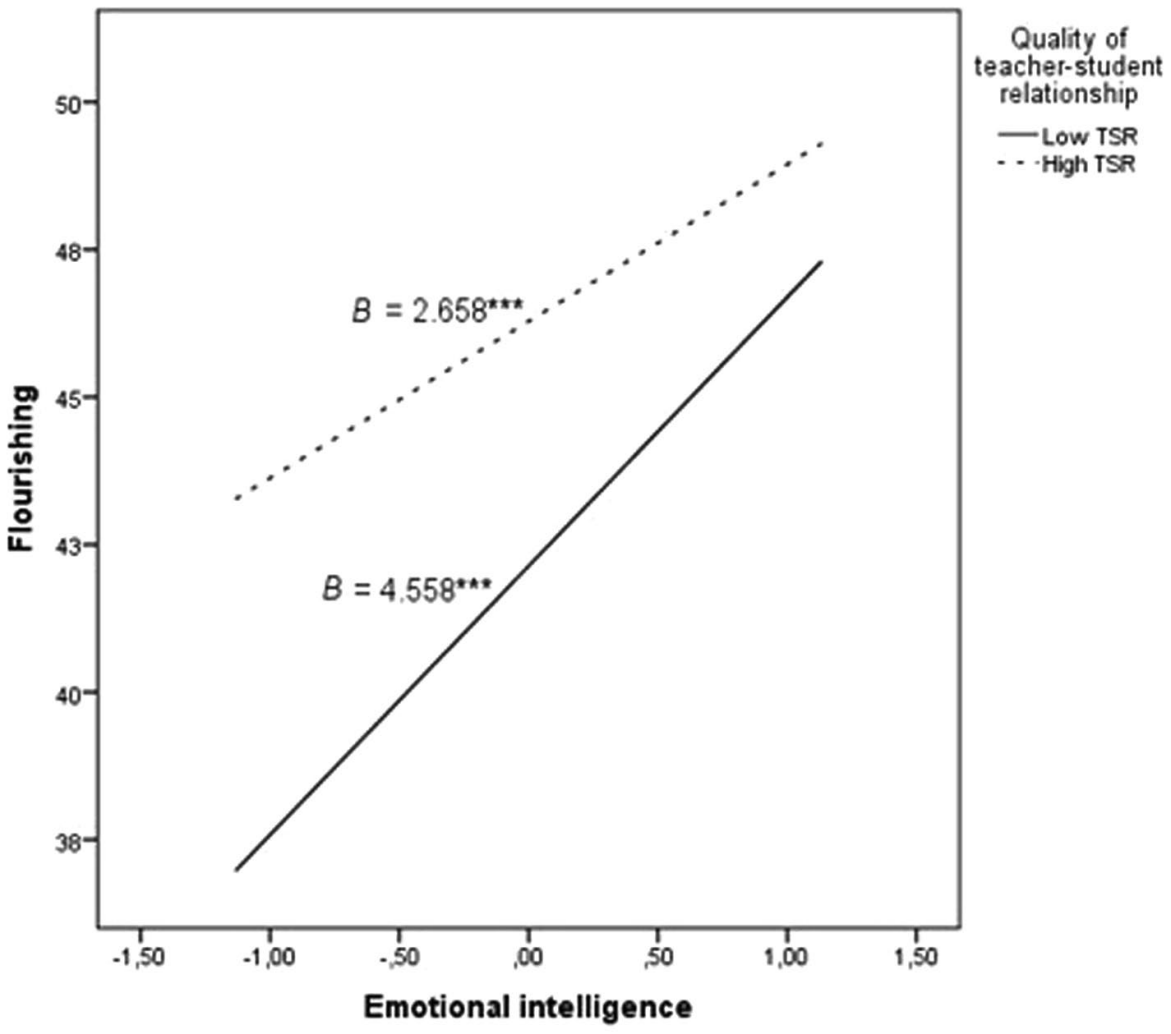
Flourishing as a function of emotional intelligence at low (*M* − SD) and high (*M* + SD) levels of teacher-student relationship quality. *B* = unstandardized coefficients; ^***^p < 0.001.

Model 7 of the PROCESS macro (Hayes, 2018) was used to test if the quality of teacher-student relationship moderated the previous mediation model. As shown in Table 3, despite emotional intelligence and teacher-student relationship being positively associated with flourishing (*p* < 0.001), their interaction was negatively related to this outcome variable. Figure 1 illustrates this effect at two levels of the moderator: low (*M* – SD) and high (*M* + SD) teacher-student relationship. As presented, in adolescents with low teacher-student relationship, the association between emotional intelligence and flourishing is stronger, suggesting that the quality of teacher-student relationship is particularly important in adolescents with low levels of emotional intelligence to predict their flourishing. Furthermore, the conditional indirect effect of emotional intelligence on academic performance through flourishing was obtained at these two levels of teacher-student relationship. The lower part of Table 3 shows 95% bootstrap CI and the index of moderated mediation, which indicate that this effect was significantly different from zero. Thus, teacher-student relationship moderated the association between emotional intelligence and flourishing, which mediated and positively predicted academic performance, confirming hypotheses 1 and 2.

**Table 3:** The indirect effect of emotional intelligence on academic performance through flourishing conditioned by teacher-student relationship quality.

Predictors	On flourishing				On academic performance			
	*B*	SE	*t*	*p*	*B*	SE	*t*	*p*
Constant	42.889	4.331	9.902	<0.001	10.054	1.558	6.452	<0.001
Gender (cov)	1.507	0.661	2.279	0.023	0.193	0.203	0.952	0.341
Age (cov)	−0.064	0.297	−0.216	0.828	−0.453	0.090	−5.024	<0.001
Emotional intelligence	3.608	0.315	11.428	<0.001	0.006	0.116	0.052	0.958
Teacher-student relationship	3.689	0.653	5.649	<0.001				
EI × TSR	−1.688	0.512	−3.294	0.001				
Flourishing					0.042	0.017	2.385	0.017
*R*^2^	0.499				0.133			
*F*	52.125			<0.001	10.066			<0.001
Conditional indirect effect of EI on AP through flourishing at levels of TSR								
	Boot Indirect effect		Boot SE		Boot LLCI		Boot ULCI	
*M* − SD	0.192		0.082		0.042		0.359	
*M* + SD	0.112		0.059		0.022		0.257	
	Index of moderated mediation		Boot SE		Boot LLCI		Boot ULCI	
	−0.071		0.037		−0.152		−0.009	

*N* = 267; *B*, unstandardized coefficient; SE, standard error; cov, covariate; EI, emotional intelligence; TSR, teacher-student relationship. Gender was dummy coded (1 = male, 2 = female). LL, lower limit; UL, upper limit; CI, confidence interval.

## Discussion

The present study used a moderated mediation to investigate whether flourishing would mediate the link between emotional intelligence and academic performance, and whether teacher-student relationship would moderate the association between emotional intelligence and academic performance in a sample of adolescents. Our results are in accordance with previous studies underlying the key role of emotional abilities on some educational outcomes such as academic performance (e.g., MacCann et al., 2020; Sánchez-Álvarez et al., 2020).

Regarding our first hypothesis, the results corroborated the mediator role of flourishing in the relation between emotional intelligence and academic performance. In line with previous research (Sánchez-Álvarez et al., 2016; Rey et al., 2019; Trigueros et al., 2019), these findings suggest that emotionally intelligent adolescents report higher levels of well-being and psychological functioning (i.e., flourishing). In addition, these higher levels of flourishing seem to be linked to higher reported academic performance (Datu, 2018; Datu et al., 2020). Thus, our contribution shows the relevance of promoting students’ flourishing as a key mechanism, which allows them to perform better in school.

With respect to our second hypothesis, the results of the moderated mediation model suggest that, despite emotional intelligence and teacher-student relationship being positively related to flourishing, their interaction seems to counterbalance their independent effects on this personal well-being indicator. Thus, developing emotional intelligence skills are a crucial factor in fostering flourishing in adolescents, especially if they have a poor relationship with their teachers. Previous studies have found that positive teacher-student relationship contributes greatly to adolescents’ adjustment and well-being (e.g., Bakadorova and Raufelder, 2018; Lan and Moscardino, 2019; Borraccino et al., 2020; Dong et al., 2021). Our results expand on these findings by suggesting that, when the quality of the relationship with teachers is low, the association between emotional intelligence and flourishing become stronger.

The present study is not without limitations. Firstly, we used cross-sectional data, which does not allow draw any causal inferences. Future studies should use longitudinal designs to clarify causal directionality among personal (i.e., emotional intelligence) and social resources (i.e., teacher-student relationship) on flourishing and academic performance. Therefore, it would be important in further research to investigate the extent to which levels of emotional intelligence, flourishing, and teacher-student relationship predict changes in academic performance in adolescents across time. Another limitation of the study is that it relied on self-reported measures of academic achievement, which could be subject to social desirability bias or memory issues. Although this measurement was taken to guarantee anonymity and was tend to be a reliable indicator (Kuncel et al., 2005), ideally future research should examine the effect of actual grade point average. Thirdly, when assessing teacher-student relationship, we only measured students’ perspective, so future studies should evaluate teachers’ point of view to ensure a more comprehensive approach of this variable.

Despite these limitations, our study is the first to analyze flourishing as an underlying mechanism that explaining the association between emotional intelligence and academic performance in adolescents and teacher-student relationship as a moderator in this relationship. These findings have important practical implications. Positive psychology’s goal is to build human flourishing, which results from the experience of positive emotions, engagement, positive relationships, meaning, and accomplishments (Seligman, 2011). Our results support the notion that interventions aiming at promoting the different dimensions of flourishing may not only have an impact on adolescents’ general well-being but also specifically on their academic performance (e.g., Van Zyl and Stander, 2019). Moreover, several reviews and meta-analyses provide evidence to consider emotional intelligence as a trainable ability in adults (Hodzic et al., 2018; Kotsou et al., 2019; Mattingly and Kraiger, 2019). Nonetheless, some intervention programs also have found that emotional intelligence can be trained in adolescents (e.g., Motamedi et al., 2017; Viguer et al., 2017; Cantero et al., 2020). In line with our findings, researchers and practitioners should foster the development of students’ emotional intelligence, particularly among those who have low-quality relationships with their teachers. Lastly, our results also imply that for adolescents with a good teacher-student relationship, emotional intelligence positively predicts flourishing to a lesser degree, so positive teacher-student relationship should also be fostered as a personal resource to improve adolescents’ flourishing and academic performance.

In sum, our study provides some empirical evidence to support the importance of developing personal and social resources (i.e., emotional intelligence and teacher-student relationship) to foster adolescents’ well-being and improve their academic performance.

## Data Availability Statement

The raw data supporting the conclusions of this article will be made available by the authors, without undue reservation.

## Ethics Statement

The studies involving human participants were reviewed and approved by the Ethical Committee of University of Málaga. Written informed consent to participate in this study was provided by the participants’ legal guardian/next of kin.

## Author Contributions

All authors listed have made a substantial, direct, and intellectual contribution to the work, read, and approved it for publication.

### Conflict of Interest

The authors declare that the research was conducted in the absence of any commercial or financial relationships that could be construed as a potential conflict of interest.
